# Study on the active ingredients and mechanism of action of Jiaotai Pill in the treatment of type 2 diabetes based on network pharmacology: A review

**DOI:** 10.1097/MD.0000000000033317

**Published:** 2023-03-31

**Authors:** Xiaona Chen, Zhao Yang, Lin Du, Yuxin Guan, Yunfang Li, Chenggang Liu

**Affiliations:** a Hei Long Jiang University of CM, Harbin, China.

**Keywords:** action target, active component, Jiaotai Pill, network pharmacology, type 2 diabetes

## Abstract

To explore the potential active ingredients and related mechanisms of Jiaotai Pill in the treatment of Type 2 diabetes mellitus (T2DM) based on network pharmacology and molecular docking. The main active components of Jiaotai Pills were obtained by TCMSP and BATMAN-TCM database combined with literature mining, and the targets of the active components of Jiaotai Pills were predicted by reverse pharmacophore matching (PharmMapper) method. Verifying and normalizing the obtained action targets by using a Uniprot database. Obtaining T2DM related targets through GeneCards, the online mendelian inheritance in man, DrugBank, PharmGKB and therapeutic target databases, constructing a Venn diagram by using a Venny 2.1 online drawing platform to obtain the intersection action targets of Jiaotai pills and T2DM, and the protein–protein interaction network was constructed by String platform. Bioconductor platform and R language were used to analyze the function of gene ontology and the pathway enrichment of Kyoto Encyclopedia of Genes and Genomes. A total of 21 active components and 262 potential targets of Jiaotai Pill were screened by database analysis and literature mining, including 89 targets related to T2DM. Through gene ontology functional enrichment analysis, 1690 biological process entries, 106 molecular function entries and 78 cellular component entries were obtained. Seven pathways related to T2DM were identified by Kyoto Encyclopedia of Genes and Genomes pathway enrichment analysis. Jiaotai Pill can achieve the purpose of treating T2DM through multiple active ingredients, multiple disease targets, multiple biological pathways and multiple pathways, which provides a theoretical basis for the clinical treatment of T2DM by Jiaotai Pill.

## 1. Introduction

Diabetes mellitus (DM) is a metabolic disorder syndrome caused by the absolute or relative insufficiency of insulin secretion and the decrease of insulin sensitivity of the corresponding target tissues. It is characterized by hyperglycemia. Long-term hyperglycemia can cause multiple organ damage, such as cardiovascular and cerebrovascular, kidney, nervous system and eye.^[1]^ The Epidemiological survey of diabetes mellitus conducted by the Endocrinology Branch of the Chinese Medical Association in 31 provinces in China from 2015 to 2017 showed that the prevalence of diabetes mellitus among people aged 18 and above in China was 11.2%,^[2]^ and the main type of diabetes was Type 2 DM (T2DM). The incidence of T2DM is about 90%, and T2DM is often accompanied by metabolic syndrome such as hypertension, dyslipidemia and obesity. As a result, the risk and harm of complications of T2DM are significantly increased, and modern people are not alert to the harm of T2DM.^[3]^ At present, the preferred treatment for T2DM is western medicine, but long-term use of western medicine will lead to adverse reactions, such as liver and kidney damage, hypoglycemia or obesity in mild cases, and shock and cardiovascular and cerebrovascular diseases in severe cases. Therefore, modern medicine proposes that the treatment strategy for T2DM is comprehensive, including the control of blood sugar, blood pressure, blood lipids and weight.^[4]^ Modern pharmacological studies have shown that some traditional Chinese medicines (TCM) are rich in hypoglycemic active ingredients such as alkaloids, flavonoids, terpenoids and polysaccharides, which can be used to treat T2DM and reduce and improve its complications.^[5]^ In view of the multi-organ targeted damage of T2DM, the TCM compound has the advantages of multiple active ingredients and extensive effects, which can solve this problem. The TCM compound ingredients can intervene in the development and treatment of the disease through multiple ways, such as adding single target effect, synergistic effect on multiple targets and toxicity dispersion effect, and can effectively play a role in clinical treatment of reducing toxicity and enhancing efficacy.^[6]^

The prescription and name of Jiaotai Pill are clearly recorded in Wang Shixiong, a famous doctor in the Qing Dynasty, in the chapter of “Brief Prescriptions of Four Departments, Anshen,” “Shengchuan Lian Wuqian, Cinnamon Heart Wufen. Jiaotai Pill is widely used in clinical practice. Besides the treatment of insomnia, modern physicians often use Jiaotai Pill to treat T2DM, nonalcoholic fatty liver and other diseases. Rhizoma Coptidis was first recorded in Shen Nong’s Classic of Materia Medica. It is bitter in taste and cold in nature. It has the effects of clearing heat and drying dampness, purging fire and detoxifying. Modern pharmacology has proved that Rhizoma Coptidis can reduce the blood sugar level of the body through its antioxidant effect.^[7]^ The cinnamon is pungent and sweet in taste and hot in nature, and has the effects of tonifying fire and supporting Yang, inducing fire to return to the primordial qi, dispelling cold and relieving pain, warming and dredging meridians, Modern pharmacological studies have shown that cinnamon can increase insulin sensitivity, improve the secretion level of glucagon-like peptide-1, further delay gastric emptying, and inhibit the activity of intestinal α-glucosidase to control the blood glucose level of T2DM patients.^[8]^ Modern studies have found that Jiaotai Pill is effective in the treatment of diabetes mellitus, and its mechanism of action is diverse, including the protection of islet B cells, the promotion of insulin secretion, and the improvement of IF. In addition, Jiaotai Pill has outstanding performance in the prevention and treatment of T2DM complications.^[9]^ However, the molecular mechanism of Jiaotai Pill is still unclear due to the numerous chemical components of TCM and the complex and diverse regulatory effects on the target, which is also one of the problems to be solved by researchers.

As a new mode of modern TCM research and development, network pharmacology of TCM contains the holistic concept of TCM, including the unique diagnosis and treatment ideas of TCM and rich clinical experience, which constitutes a research mode characterized by “network and “system.”^[10]^ The network pharmacology of TCM clarifies the mechanism of action of drugs and their components by constructing the network of active ingredient-target, active ingredient-target-disease, active constituent-target-action pathway of TCM, which is suitable for the study of the mechanism of action of compound Chinese medicine.^[11]^

Traditional Chinese Medicine Systems Pharmacology Database and Analysis Platform (TCMSPDA) TCMSP is a platform and database for Chinese medicine research based on systems pharmacology, which contains the key information of Chinese herbal medicine in the Chinese Pharmacopoeia, such as composition, molecular structure, target of action and related diseases. It provides a basis for screening the active ingredients of TCM, studying the mechanism of drug action, broadening the therapeutic direction of ancient prescriptions and building new compound compatibility, and provides a new way for modern prescriptions and TCM research.^[12]^ Therefore, it is of great significance to screen the main active ingredients of Jiaotai Pill and explore its targets for the treatment of T2DM by means of network pharmacology, and to analyze the molecular mechanism of Jiaotai Pill in the treatment of T2DM by constructing the biological functions and pathway networks involved in the active ingredient-target, protein interaction, protein molecular docking and targets.

## 2. Materials and methods

### 2.1. Collection of main chemical components of Jiaotai Pill

Using TCMSP (https://old.tcmsp-e.com/tcmsp.php) database and BATMAN-TCM (a Bioinformatics Analysis Tool for Molecular mechANism of Traditional Chinese Medicine) Database (http://bionet.ncpsb.org/batman-tcm/)^[13]^ and other ways to collect the main chemical components of Coptis chinensis and Cinnamomum cassia, which constitute Jiaotai Pill, and then combined with literature data mining and data integration, the main chemical components of Coptis chinensis and Cinnamomum cassia were screened out according to the content of the components in the drug and related biological functions.

### 2.2. Excavation and screening of active ingredients of Jiaotai Pill

After oral administration, TCM needs to go through the process of absorption, distribution, metabolism and excretion to reach the target site and play a therapeutic role. Oral bioavailability (OB) is one of the most important pharmacokinetic parameters in absorption, distribution, metabolism and excretion. OB is an important index to determine the drug likeness (DL) index of active ingredients. Substances with OB ≥ 30% are considered to have high bioavailability; DL is used to estimate molecular druggability in drug design, and its index can be used for rapid screening of active substances, and substances with DL index ≥ 0.18 are considered to be highly druggable.^[14]^ Therefore, in the TCMSP database, the screening conditions were set as the chemical components with OB ≥ 30% and DL ≥ 0.18 as the candidate active components. The active ingredient of that Jiaotai Pill is finally obtain by screening by set the threshold values Score cutoff of a drug-target similarity model to be more than or equal to 20 and P to be less than or equal to 0.05 in the BATMAN-TCM database, The molecular structures were further confirmed by TCMSP and PubChem (https://pubchem.ncbi.nlm.nih.gov/) platform, and finally the compounds were saved as mol2 format files.

### 2.3. Obtain the target of Jiaotai Pill and the target related to T2DM

The virtual protein targets were obtained by logging in the PharmMapper server^[15]^ and uploading the mol2 (*.mol2) format files of the screened active ingredients of Coptis chinensis and Cinnamomum cassia. The selected virtual protein targets of Jiaotai Pill were mined for gene target information, and UniProt database^[16]^ (https://www.uniprot.UniProtKB search function in org/), input the screened protein molecules, the database selection is limited to “Reviewed (Swiss-Prot),” the species is “Human,” query the target, and obtain the gene target of the active ingredient of Jiaotai Pill. The screening of T2DM disease targets was carried out by Gene cards (The Human Gene Database) (https://www.genecards./), online Mendelian inheritance in man database (https://www.omim.org/), Drug (DrugBank) database (https://go.drugbank.com/), Global pharmacogenomic information resource PharmGKB Database^[17]^ (https://www.pharmgkb.org/), therapeutic Target Database^[18]^ (http://db.Idrblab.Net/TTD/), and the higher the Relevance score in the Gene cards database, the closer the relationship between the target and the disease, so the target with Relevance score greater than the median was taken as the target of T2DM.^[19]^ The reported genes related to T2DM were searched by inputting the keyword “type 2 diabetes mellitus,” and the duplicate and false positive genes were excluded. Verified by literature retrieval, the finally obtained T2DM related target is matched with the Jiaotai Pill action target obtained by PharmMapper, so as to obtain the potential hypoglycemic action target of the Jiaotai Pill active ingredient. Through the online software Venny 2.1 mapping platform^[20]^ (https://bioinfogp.cnb.csic.es/tools/venny/index). The Venny plots of T2DM target screening and active ingredient-T2DM target of Jiaotai Pill were drawn respectively.

### 2.4. Construction of active ingredient-T2DM target network model and protein-protein interaction network of Jiaotai Pill

#### 2.4.1. Construction and analysis of network model of key active ingredient-T2DM target of Jiaotai Pill.

The information of active components and targets of Jiaotai Pill was imported into Cytoscape V3.9.0 software, and the interaction network diagram of “Jiaotai Pill-active components-T2DM-targets” was constructed. Network Analyzer of Cytoscape V3.9.0 software was used to calculate the constructed network graph, and the degree of the network was analyzed by statistical analysis to screen out the core active components.

#### 2.4.2. Construction of protein-protein interaction network.

The target of Jiaotai Pill was introduced into the database of functional protein association networks^[21]^ (https://cn.string-db). Homo sapiens was defined as the species by using the multiple proteins tool, and the protein interaction was obtained. During the acquisition process, the minimum interaction threshold of the protein target was set as “highest confidence” (>0.9), and other settings were default settings. Hide the free protein in the result and save the TSV format file. The information of node1 and node2 in the file was imported into Cytoscape V3.9.0 software to draw the protein-protein interaction network, and the topology of the network was analyzed. At the same time, researchers found that the MCC algorithm of Cytohubba plug-in of Cytoscape V3.9.0 software has outstanding advantages in predicting disease targets.^[22]^ Therefore, in this study, the MCC algorithm was used to screen the core targets from the potential target data, and the core target data was visualized to construct the protein–protein interaction (PPI) network diagram.

### 2.5. Analysis of biological processes and metabolic pathways of core targets

The Bioconductor platform (http://bioconductor.org/biocLite.R) was used to query the ID of the related core target genes of Jiaotai Pill-T2DM. Install the Bioconductor platform with R language tools, adjust the parameters *P* and *q* value to <0.05, and then perform gene ontology (GO) on the core targets. GO and Kyoto Encyclopedia of Genes and Genomes (KEGG) enrichment analysis, selection of biological process (BP), cell component (CC). The 3 modules of CC and molecular function (MF) were used for GO enrichment analysis, and KEGG was used for pathway analysis. The pathways that were related to the pathogenesis of T2DM and enriched with most of the key genes were selected for target-pathway analysis, and the relevant pathway maps were drawn.

## 3. Results

### 3.1. Screening of active ingredients in Jiaotai Pills

The active components of the Coptis and the cinnamon are screened by setting the screening conditions of a TCMSP database as that the OB is more than or equal to 30% and the DL is more than or equal to 0.18 and setting a BATMAN database as that the Score cutoff is more than and equal to 20 and the *P* is less than or equal to .05; in addition, data mining of related medicine active components is carried out in published and proved literatures. According to the content and biological function of the active components, 21 active components were selected from Jiaotai Pills, including 13 components from Coptis chinensis and 8 components from Cinnamomum cassia. In addition, the content of cinnamaldehyde and cinnamic acid in cinnamon is high, but its OB and DL values are low. According to the screening principle, cinnamaldehyde and cinnamic acid are included in the active ingredients, as shown in Table 1.

**Table 1:** Main active ingredients and some pharmacokinetic parameters of Jiaotai Pill.

Traditional Chinese Medicine	Active ingredient	OB (%)	DL
Coptis Chinensis	Wore nine	45.83	0.87
	Coptisine	30.67	0.86
	Berlambine	36.68	0.82
	Berberine	36.86	0.78
	Epiberberine	43.09	0.77
	Hydrogenated berberine ((R) -Canadine)	55.37	0.77
	Obacunone	43.29	0.77
	Berberrubine	35.74	0.72
	Jatrorrhizine	30.44	0.75
	Rhubarb dianthrone A (Palmidin A)	35.36	0.65
	Palmatine	64.60	0.64
	Quercetin	46.43	0.28
	Moupinamide	86.71	0.26
Cinnamon	Procyanidin B1	67.87	0.66
	Stigmasterol	43.83	0.76
	Quercetin	46.43	0.28
	Cinnamaldehyde	31.99	0.02
	Cinnamic acid	19.68	0.03
	β-sitosterol	36.91	0.75
	Kaempferol	41.88	0.24
	(-) -epigallocatechin-3-gallate	55.09	0.77

### 3.2. Prediction of the active ingredients of Jiaotai Pill and the target of T2DM intervention

**Figure 1.**
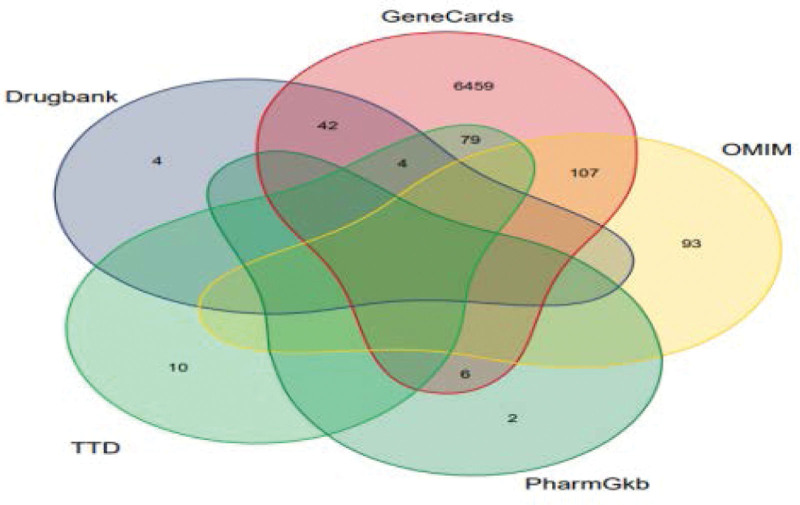
Venn diagram of T2DM target screening. T2DM = type 2 diabetes mellitus.

**Figure 2.**
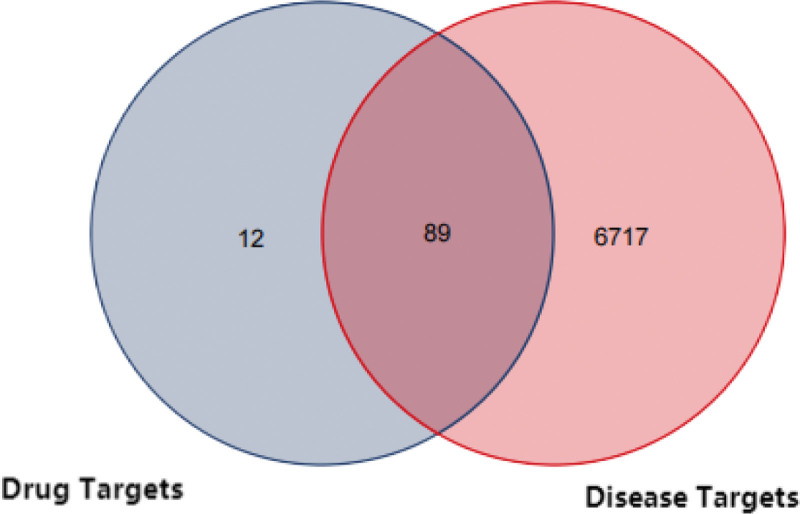
Venn diagram of active ingredient of Jiaotai Pill and potential target of T2DM. T2DM = type 2 diabetes mellitus.

The top 300 potential targets of 21 core active ingredients of Jiaotai Pill returned in PharmMapper were sorted from high to low according to Normalized Fit Score, and 300 protein targets were obtained by screening to remove duplicates. By inputting the name of protein target in UniProt KB function item of Uniprot database, “Reviewed (Swiss-Prot)” and “Human” were defined, and 262 gene targets were obtained.T2DM targets were predicted in GeneCards, online Mendelian inheritance in man, DrugBank, PharmGKB, therapeutic target database and other databases, and 6717 potential targets were obtained by screening the predicted targets and removing duplicates. At the same time, the screened T2DM targets and the active ingredient targets of Jiaotai Pill were mapped with the potential targets of T2DM by Venny 2.1 online drawing tool, and Venn diagrams (Figs. 1 and 2) were drawn 0.89 core targets were obtained.

### 3.3. Construction and analysis of T2DM target network of active components of Jiaotai Pill

**Figure 3.**
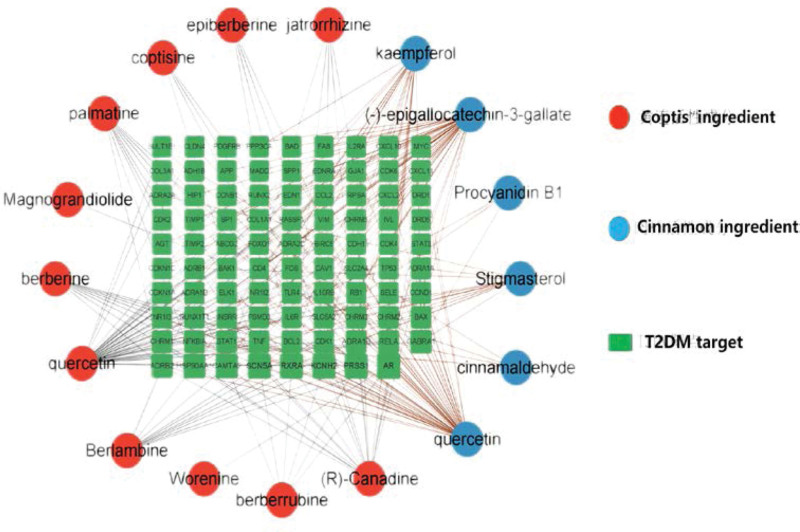
The active ingredient-T2DM target network of Jiaotai Pill. T2DM = type 2 diabetes mellitus.

The 21 active ingredients and 89 predicted targets of Jiaotai Pill were imported into Cytoscape V3.9.0 software to construct a “drug-active ingredient-T2DM-target” interaction network diagram (Fig. 3), including 105 nodes and 196 edges. The nodes are the representatives of active ingredients and action targets (the green rectangular nodes represent the action targets, and the red and blue circular nodes represent the active ingredients of Coptis chinensis and Cinnamomum cassia), and the edges are the associations between the active ingredients of drugs and the action targets of diseases (the black and brown lines represent the associations between the active components of Coptis chinensis and Cinnamomum cassia and T2DM targets). From the picture, we can see that different active ingredients can act on the same target, which reflects the characteristics of multi-component and multi-target co-action of Jiaotai Pill. The average Degree of the “Drug-active ingredient-T2DM-target” interaction network is 3.7. The active ingredients with a large degree value >7.4 were selected as the main active ingredients of Jiaotai Pill for the treatment of T2DM, including quercetin, (-) -epigallocatechin-3-gallate (EGCG), kaempferol, hydroberberine, stigmasterol, berberine extract, palmatine, berberine and so on.

### 3.4. PPI network analysis

**Figure 4.**
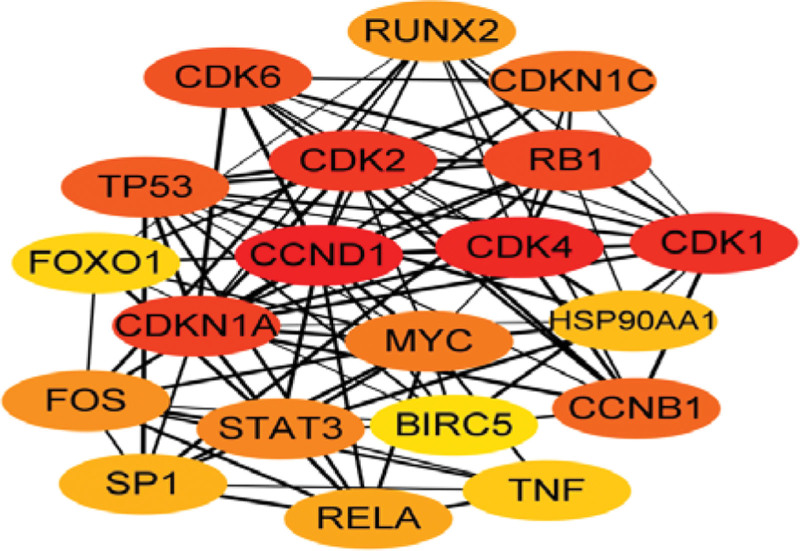
PPI network diagram. PPI = protein–protein interaction.

The obtained 89 active ingredients of Jiaotai Pill and T2DM targets were imported into the String database to obtain PPI network data. The data were imported into Cytoscape V3.9.0 software for topological analysis, and 61 nodes and 396 edges were obtained. The MCC algorithm of Cytohubba plug-in of Cytoscape V3.9.0 software was used to process the target data, and the top 20 targets with MCC values were selected to draw the PPI network diagram (as shown in Fig. 4). The larger the MCC value is, the closer the relationship between the drug and the target is. The color of the node represents the size of the MCC value, and the smaller the MCC value corresponding to the change from red to yellow. Therefore, the 20 targets obtained are taken as the core targets of Jiaotai Pill in the treatment of T2DM, as shown in Table 2.

**Table 2:** Core targets of Jiaotai Pill for T2DM.

Serial number	Gene target	Core protein target	MCC value
1	*CCND1*	G1/S-specific cyclin D1 (G1/S-specific cyclin-D1)	3426
2	*CDK4*	Cyclin-dependent kinase 4	3384
3	*CDK1*	Cyclin-dependent kinase 1	3252
4	*CDK2*	Cyclin-dependent kinase 2	3006
5	*CDKN1A*	Cyclin-dependent kinase inhibitor 1	2694
6	*RB1*	Retinoblastoma-associated protein	2406
7	*CDK6*	Cyclin-dependent kinase 6	2160
8	*TP53*	Cellular tumor antigen p53	1020
9	*CCNB1*	G2/mitotic-specific cyclin-B1 (G2/mitotic-specific cyclin-B1)	846
10	*CDKN1C*	Cyclin-dependent kinase inhibitor 1C	720
11	*MYC*	Myc proto-oncogene protein	456
12	*STAT3*	Signal transducer and activator of transcription 3	282
13	*FOS*	Protein c-Fos	270
14	*RUNX2*	Runt-related transcription factor 2	246
15	*RELA*	Transcription factor p65	156
16	*HSP90AA1*	Heat shock protein HSP 90-alpha	150
17	*SP1*	Transcription factor Sp1	144
18	*BIRC5*	Baculovirus IAP repeat protein 5 (Baculoviral IAP repeat containing protein 5)	138
19	*FOXO1*	Forkhead box protein O1	132
20	*TNF*	Tumor necrosis factor	120

T2DM = type 2 diabetes mellitus.

### 3.5. Functional enrichment analysis of target GO

**Figure 5.**
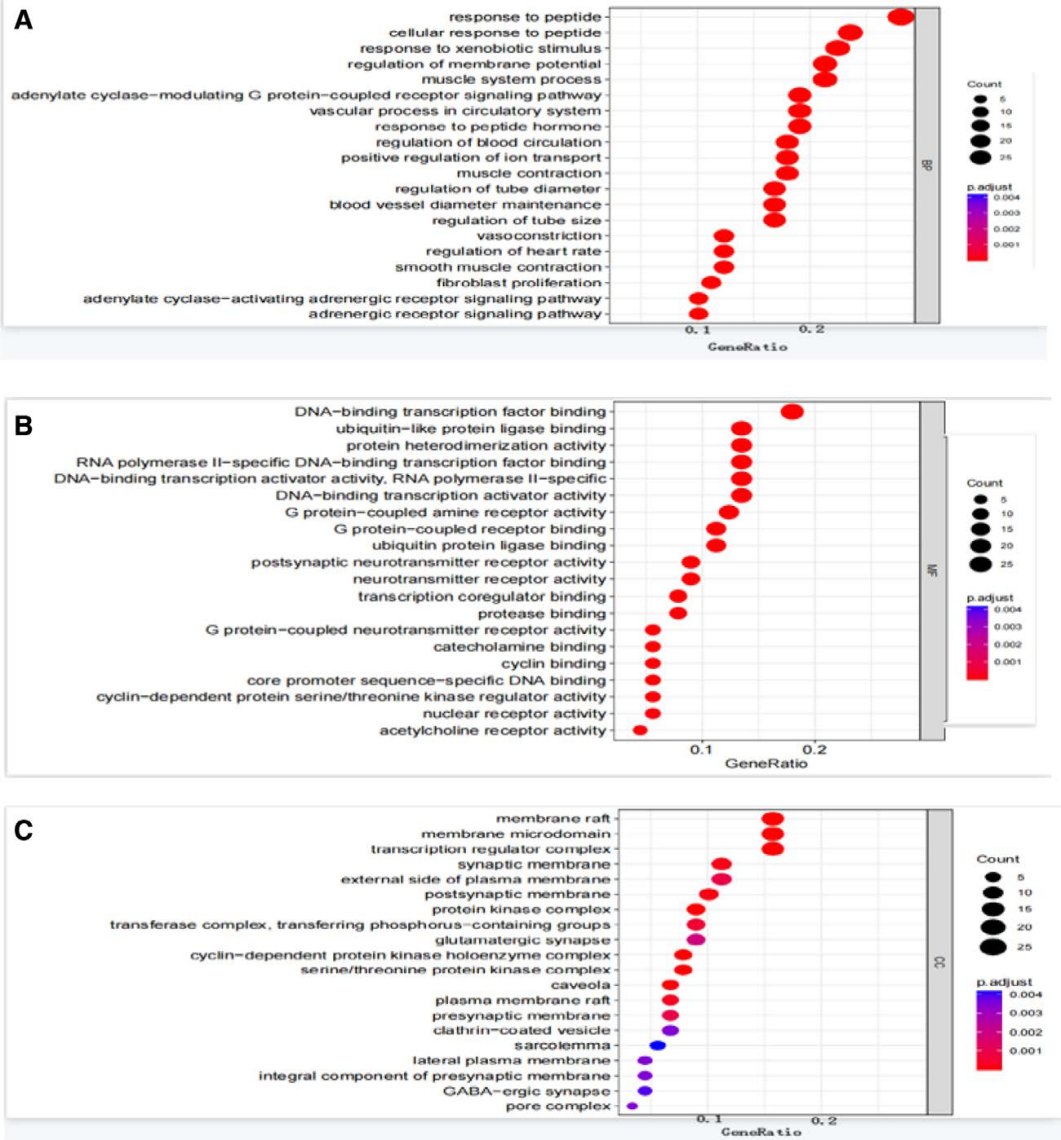
(A) Bubble diagram of GO-BA analysis. (B) GO-MF analysis bubble diagram. (C) GO-CC analysis bubble diagram. CC = cellular component, GO = gene ontology, MF = molecular function.

The 89 selected targets were analyzed by R language, and the BP, CC, and MF were selected for functional enrichment. According to the *P* value, the number of enriched genes and the ratio of genes, the first 20 data were selected according to the standard of *P* < .05. Use R language to draw bubble chart. The results showed that 1690 items were obtained by GO-BP analysis, and it was found that the BP of Jiaotai Pill in the treatment of T2DM were enriched in the response to peptide, the response of cells to peptide, the response to xenostimulation, membrane potential regulation, and muscle system process, as shown in Figure 5A. GO-MF analysis yielded 106 entries, which were found to be enriched in DNA − transcription factor binding, ubiquitin-like—protein ligase binding, protein heterodimerization, RNA polymerase II − specific DNA − binding transcription factor binding, DNA − binding transcription activator activity, RNA polymerase II − specific MF, as shown in Figure 5B. GO-CC analysis yielded 78 entries, which were found to be enriched in lipid rafts, membrane microdomains, transcription regulator complexes, synaptic membranes, plasma membrane outsides, and other CCs on the cell membrane, as shown in Figure 5C. Through GO functional enrichment analysis, the results showed that Jiaotai Pill may play a role in the treatment of T2DM by affecting the body’s response to peptides and BP in muscle, blood vessels and other systems, and by combining with target proteins such as transcription factors and enzymes to affect the synthesis of MF and CC of target proteins, mainly involving transcription factor complexes, enzyme complexes, membrane microdomains and synaptic membranes.

### 3.6. Enrichment analysis of target KEGG pathway

**Figure 6.**
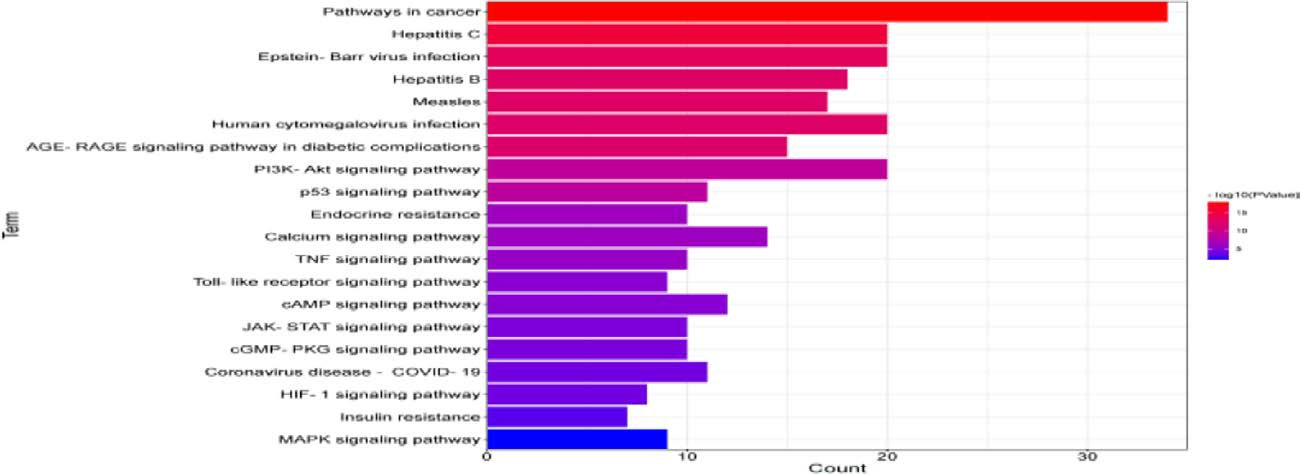
Histogram of KEGG pathway enrichment analysis. KEGG = Kyoto Encyclopedia of Genes and Genomes.

**Figure 7.**
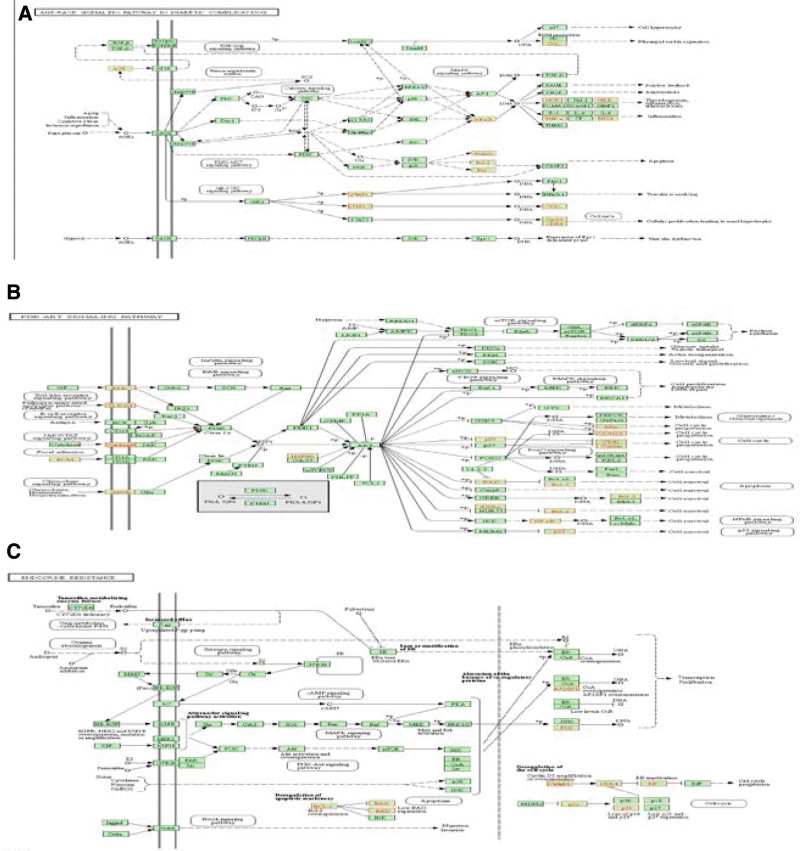
(A) Diabetes complications AGE − RAGE signaling pathway. (B) PI3K/AKT signaling pathway. (C) Endocrine resistance pathway.

KEGG pathway enrichment analysis was carried out on 89 drug and disease intersection targets obtained by screening through R language, and 124 pathway enrichment items were obtained. The results showed that the target genes of the intersection of Jiaotai Pill and T2DM were enriched in the advanced glycation end products (advanced glycation end product [AGE] − RAGE signaling pathway, phosphoinositide 3-kinase [PI3K]/AKT (protein kinase B) signaling pathway, endocrine resistance pathway, tumor necrosis factor (TNF) signaling pathway and Toll-like receptor signaling pathway are shown in Figure 6. At the same time, the pathway mechanism map related to T2DM was drawn through the KEGG Mapper platform, and the specific mechanism of Jiaotai Pill in the treatment of T2DM was further analyzed in combination with the pathway mechanism map, as shown in Figure 7.

## 4. Discussion

As a chronic metabolic disease, T2DM is characterized by hyperglycemia, insulin resistance, insufficient insulin secretion, increased hepatic glucose production and lipid metabolism disorders.^[23]^ The etiology and pathogenesis of T2DM are still unclear, but a large number of studies have shown that in addition to obesity, sedentary, high-calorie diet and population aging, IF, adipokine disorders, inflammatory response, oxidative stress and intestinal microflora abnormalities, immune disorders and mitochondrial dysfunction have become important pathological factors.^[24]^ Patients with T2DM have a higher risk of microvascular (retinal vasculopathy, renal function and peripheral neuropathy) and macrovascular (cardiovascular and cerebrovascular disease and peripheral arterial disease) complications, most organ systems are adversely affected by complications associated with T2DM, and T2DM increases the risk of cancer. At present, cancer has become one of the main causes of death in T2DM patients.^[25–27]^ At present, the main treatment strategies of T2DM are comprehensive, including the control of blood sugar, blood pressure, blood lipid and weight, antiplatelet therapy and lifestyle improvement. Medical nutrition therapy and exercise therapy belong to lifestyle management, which are the basic treatment measures to control hyperglycemia and persist for a long time. The drug treatment of hyperglycemia is to correct the impairment of IF and insulin secretion. According to the different effects, hypoglycemic drugs can be divided into drugs that promote insulin secretion and drugs that reduce blood sugar through other mechanisms.^[28]^ Western medicine hypoglycemic drugs have advantages in improving hyperglycemia-related indicators, but their way of action is single, some Western medicines have great side effects, while increasing the incidence of cancer and other diseases, the treatment results often do more harm than good. However, TCM treatment of T2DM can correct the disorder of glucose and lipid metabolism by protecting beta cells, promoting insulin secretion, improving insulin sensitivity, regulating the structure of intestinal microbiota and improving the function of immune system, which can regulate many indicators of T2DM patients and better improve their quality of life.^[29]^

The active ingredient-T2DM action target network of Jiaotai Pill (Fig. 3) found that the main active ingredients of Jiaotai Pill in the treatment of T2DM were quercetin, EGCG, kaempferol, hydroberberine, stigmasterol, berberine extract, palmatine, berberine and so on. Modern pharmacological studies have shown that the main active ingredients of Coptis chinensis are alkaloids, of which berberine (5% ~ 8%) is the most abundant alkaloid, followed by palmatine, coptisine and epiberberine, of which jatrorrhizine is relatively low.^[30,31]^ Studies have shown that protoberberine compounds, including berberine, palmatine, jatrorrhizine and berberrubine, have hypoglycemic, hypolipidemic, anti-tumor, anti-inflammatory, anti-atherosclerosis and other effects. Among them, the hypoglycemic function of berberine is mainly to promote the regeneration of islet cells, inhibit hepatic gluconeogenesis, and increase the level of glucagon-like peptide-1, serum insulin and the number of islet beta cells in the intestine.^[32–34]^ The active ingredient of Cinnamomum cassia Presl is also one of the hot researches in the treatment of diabetes in recent years. Its active ingredient EGCG has many beneficial effects such as preventing free radical damage, anticancer and antioxidant activity, improving glucose and lipid metabolism and protecting cardiovascular system.^[35]^ Meanwhile, foreign scholars have proved that EGCG can improve IF and enhance insulin sensitivity. Can control lipid metabolism, reduce body weight and that like, ECGC has a broad prospect in the treatment of type 2 diabetes.^[36]^ Kaempferol and quercetin, which belong to the same category as ECGC, are recommended as alternatives to the drug treatment of T2DM to prevent or improve T2DM and its related complications. Quercetin has anti-inflammatory, antioxidant, antihypertensive, anticancer, antiviral, and neuroprotective effects. It has strong anti-diabetic properties, can enhance the body’s glucose uptake and down-regulation of gluconeogenic enzyme activity in the liver, improve the function and proliferation of beta cells, and improve diabetic bone disease in T2DM patients. Kaempferol has anti-inflammatory, antihypertensive, lipolytic and anticancer properties, and its antidiabetic function is related to reducing pyruvate carboxylase activity, inhibiting hepatic gluconeogenesis, increasing the translocation and expression of glucose transporter 4, and improving insulin sensitivity by inhibiting proinflammatory cytokines.^[37]^ Stigmasterol also has the functions of anti-tumor, anti-inflammatory, antioxidant, cholesterol lowering and memory improvement, and also has a good anti-diabetic effect, which has potential benefits for T2DM.^[38]^ The above components have special effects on the treatment of T2DM, which indicates that the screening of the relevant active components of Jiaotai Pill in the treatment of T2DM has a reliable basis.

Using MCC algorithm of Cytoscape V3.9.0 software to screen the core targets of Jiaotai Pill in the treatment of T2DM, CCND1, CDK4, CDK1, CDK2, CDKN1A, CDK6, CCNB1, and CDKN1C in the top 10 targets are the regulators of cyclin-dependent kinases. Modern studies have found that TP53, RB1, CCNB1 and CDKN1A have anti-diabetic activity.^[39,40]^ Studies have found that CCND1 plays a role in obesity-related T2DM, which is related to its participation in fatty acid and glucose metabolic pathways and oxidation/reduction reactions^[41]^; while CDK4/6 has a regulatory effect on human beta cell proliferation.^[42]^ Studies have found that CDK1 is the key to drive oxidative phosphorylation, and the abnormal increase in the secretion of oxidative phosphorylation components may be the basis for the development of diabetes.^[43]^ In the pathogenesis of T2DM, the increase or decrease of the above targets plays an important role in the occurrence, development and treatment of T2DM.

KEGG pathway enrichment analysis was performed on the 89 selected intersection targets. It was found that the main pathways related to T2DM were age − RAGE signaling pathway, PI3K-AKT signaling pathway, endocrine resistance pathway, TNF signaling pathway and Toll-like receptor signaling pathway. Mitogen-activated protein kinase (MAPK) signaling pathway. Inhibition of PI3K-AKT signaling pathway is one of the mechanisms of IF, which is the main feature of T2DM, and chronic inflammation is also one of the important reasons leading to IF and T2DM. MAPK signaling pathway, TNF signaling pathway and Toll-like receptor signaling pathway play a vital role in inflammation, and studies have found that MAPK can regulate signaling pathways by phosphorylating target proteins, and can promote the absorption and utilization of glucose by up-regulating the expression of PI3K, AKT and glucose transporter-4. Nhance the sensitivity of the insulin receptor. Thereby improving insulin resistance, and TNF can trigger the activation of the MAPK pathway. Studies have shown that the level of TNF receptors in the circulatory system can predict the development of diabetes and the occurrence of complications.^[44,45]^ Second, the AGE-RAGE axis is activated by signals transducing oxidative stress and inflammatory responses that damage vascular endothelial cells, renal mesangial/endothelial/smooth muscle/podocytes, and retinal pericytes, leading to the diabetic triad. Hyperglycemia promotes the formation of AGEs, and proximal tubular injury delays the catabolism and clearance of AGEs. This forms a vicious circle in the development of T2DM, but studies have confirmed that flavonoids and phytochemicals can act as AGE-forming inhibitors to block the AGE-RAGE signaling pathway. It has also been found that flavonoids enhance glucose uptake in peripheral metabolic tissues by protecting and proliferating pancreatic beta cells, improving their insulin secretion function, and improving the efficacy of insulin in preventing and alleviating obesity and diabetes by inhibiting inflammation, lipotoxicity, and oxidative stress.^[46,47]^ The active ingredients of Jiaotai Pill act on the pathways mentioned above, on the 1 hand, it can inhibit the damage of inflammatory factors to pancreatic beta cells, and promote the proliferation of pancreatic beta cells, on the other hand, it can improve if and enhance insulin sensitivity. It can control lipid metabolism, reduce body weight and other multiple effects, and also has a good preventive effect on T2DM complications.

Modern studies have found that gut microbial-derived metabolites can affect host obesity, insulin resistance and hormone secretion, thus jointly affecting the progression of T2DM. The regulation of insulin secretion is controlled by nutrients (such as glucose, free fatty acids), endocrine peptides, hormones (such as insulin, incretin, growth hormone, prolactin, glucagon, etc.) and autonomic nervous system.^[48]^ Moreover, intestinal flora can promote the absorption of active ingredients of Chinese herbal medicine. For example, the oral availability of quercetin is low, but some enzymes in intestinal bacteria and intestinal mucosal epithelial cells can convert quercetin and its derivatives into various metabolites such as phenolic acids, which are absorbed, transformed or excreted by the intestine.^[49]^ Researchers found that Jiaotai Pill could inhibit the secretion of inflammatory cytokines TNF-α and IL-6, promote the secretion of intestinal flora metabolites, and further promote the secretion of insulin. Therefore, the development prospect of Chinese herbal medicine in the treatment of T2DM should focus on the function of intestinal flora.^[50]^

To sum up, this study studied the mechanism of Jiaotai Pill in the treatment of T2DM by network pharmacology, and found that the active ingredients of Jiaotai Pill may be quercetin, EGCG, kaempferol, hydroberberine, stigmasterol, berberine, palmatine and so on. Combine with T2DM target proteins such as CCND1, CDK4, CDK1, CDK2, CDKN1A, RB1, CDK6, TP53, CCNB1 and CDKN1C, And further regulate that AGE-RAGE signal pathway, the PI3K-AKT signal pathway, the endocrine resistance pathway, the TNF signal pathway and the Toll-like receptor signal pathway as well as the insulin resistance pathway and the MAPK signal pathway of diabetic complications. Inhibition of T2DM formation through improvement of IF, enhancement of insulin sensitivity, inhibition of inflammatory response and oxidative stress and enhancement. The results show that Jiaotai Pill can treat T2DM through multiple active ingredients, multiple disease targets, multiple biological pathways and multiple pathways, which provides a theoretical basis for the clinical treatment of T2DM and a basis for broadening the clinical use of Jiaotai Pill.

## Author contributions

**Conceptualization:** Xiaona Chen, Chenggang Liu.

**Data curation:** Xiaona Chen, Chenggang Liu.

**Formal analysis:** Xiaona Chen, Chenggang Liu.

**Funding acquisition:** Xiaona Chen.

**Investigation:** Xiaona Chen.

**Methodology:** Xiaona Chen.

**Project administration:** Xiaona Chen.

**Resources:** Xiaona Chen, Lin Du.

**Software:** Xiaona Chen, Chenggang Liu.

**Supervision:** Xiaona Chen.

**Validation:** Xiaona Chen, Zhao Yang.

**Visualization:** Xiaona Chen.

**Writing – original draft:** Xiaona Chen, Zhao Yang, Lin Du, Yuxin Guan, Yunfang Li, Chenggang Liu.

**Writing – review & editing:** Xiaona Chen, Zhao Yang, Lin Du, Yuxin Guan, Yunfang Li, Chenggang Liu.
